# “You have to keep your nerve on a DMC.” Challenges for data monitoring committees in neonatal intensive care trials: qualitative accounts from the bracelet study

**DOI:** 10.1186/1745-6215-16-S2-O38

**Published:** 2015-11-16

**Authors:** Claire Snowdon, Diana Elbourne, Peter Brocklehurst, Martin Ward Platt, Robert Tasker

**Affiliations:** 1 London School of Hygiene and Tropical Medicine, University of London, London, UK; 2 University College London, London, UK; 3 Royal Victoria Infirmary, Newcastle upon Tyne, UK; 4 Harvard Medical School, Boston, MA, USA; 5 London Hub for Trials Methodology Research, London, UK

## Background

Data Monitoring Committees (DMCs) are essential to the good conduct of many trials. Typically they comprise a small expert group which monitors safety, efficacy, progress and early outcome data as trials recruit. DMCs can recommend protocol revisions and early stopping of a trial. As DMC meetings usually consider unblinded interim data confidentially, they are seldom exposed to research scrutiny. An important exception was case studies presented in the DAMOCLES project.

## Methods

Using interviews with participants in the BRACELET Study on bereavement in neonatal intensive care trials, qualitative accounts of experiences of 18 DMC members were used to build on these earlier case studies.

## Results

Interviewees considered the neonatal research populations especially vulnerable, and that outcomes that included both death and major disability were crucial for families. DMC members discussed particular difficulties of balancing the competing risks of these outcomes, especially when mortality data were available long before data on longer term disability could be collected. An additional consideration was the choice of age at which disability was assessed, as some manifestations would not become apparent in the short term. Interviewees commonly used the imagery of bravery, and described DMCs either holding or losing their nerve.

## Conclusions

DMCs for trials in other fields may also face difficult ethical trade-offs in monitoring composite outcomes. The experience from this sample of DMC members suggest that, for neonatal trials, the combination of the importance of the timing of one component of the outcome assessment, alongside the vulnerability of the population, presented particular challenges.

